# “We’re so limited with what we actually can do if we follow all the rules”: a qualitative study of the impact of COVID-19 public health protocols on violence against women services

**DOI:** 10.1186/s12889-022-13550-w

**Published:** 2022-06-13

**Authors:** C. Nadine Wathen, Caitlin Burd, Jennifer C. D. MacGregor, Jill Veenendaal, Isobel McLean, Tara Mantler, the Violence Against Women Services in a Pandemic Research Team

**Affiliations:** 1 Arthur Labatt Family School of Nursing, Western University, ON N6A 5B9 London, Canada; 2 Faculty of Information & Media Studies, Western University, ON London, Canada; 3 School of Architecture & Landscape Architecture, University of British Columbia, 402 - 6333 Memorial Road, BC V6T 1Z2 Vancouver, Canada; 4 School of Health Studies, Western University, ON London, Canada

**Keywords:** Violence against women, Domestic violence, Shelters, COVID-19, Pandemic, Public health regulations, qualitative research

## Abstract

**Background:**

Violence against women (VAW) is a major public health problem that grew worse during the COVID-19 pandemic. While all services were impacted by changing pandemic guidance, VAW shelters, as congregate settings with multiple funders and regulators, faced unique challenges.

**Methods:**

We conducted a qualitative analysis of interviews with 26 women’s shelter staff and eight women accessing care, as well as 10 focus groups (five each at two time points approximately a year apart) involving 24 leaders from VAW and related services in Ontario, Canada.

**Results:**

We identified eight overlapping themes specific to government and public health COVID-19 regulations and their application in women’s shelters. Overall, inconsistency or lack of clarity in rules, and how they were communicated, caused significant stress for women using, and staff providing, services. Staff and leaders were very concerned about rules that isolated women or replicated other aspects of abusive relationships. Women wanted to understand what options were available and what was expected of them and their children in these spaces. Leaders sought clarity and consistency from their various government funders, and from public health authorities, in the face of ever-evolving directives. As in the broader public, there was often the perception that the rules did not apply equally to everyone, for example, for women of colour using VAW services, or those whose first language was not English.

**Conclusions:**

In the absence of consistent pandemic guidance and how to implement it, many VAW services devised tailored solutions to balance safety from COVID-19 with women’s physical and emotional safety from abuse and its impacts. However, this was difficult and exhausting. A key policy implication is that women’s shelters are a distinct form of congregate housing; they are very different in terms of services provided, size, type and age of facilities from other congregate settings and this must be reflected in public health directives. Better communication and synchronization of policies among government funders and public health authorities, in consultation with VAW sector leaders, would mean protocols tailored to minimize harm to women and children while protecting health and safety.

## Background

Violence against women (VAW) is a major public health crisis in Canada and globally [1]. As noted early in the COVID-19 pandemic, times of crisis are known to make VAW worse, both in incidence and severity [2]. In fact, in March 2020, the United Nations (UN) Secretary General predicted a “horrifying global surge in domestic violence” [3]. This proved to be prescient, with rates of VAW rising globally, one in two women reporting that they or a woman they know experienced violence during the pandemic, and seven in 10 women feeling that domestic violence increased [4]. In Canada, before the pandemic, a woman was killed by a current or former partner about once a week and a woman or girl killed every 2.5 days. The most recent data in Canada shows an increase to one femicide approximately every 1.5 days [5, 6].

Many inter-related factors are associated with these increases in violence during the pandemic, including, as summarized by the World Health Organization [7], disruption to services and access to formal and informal support networks, and lack of availability of safe shelter due to service restrictions. Along with a range of relationship and pandemic specific factors (e.g., lockdowns bringing increased proximity to the abuser with no ability for respite or escape and increased financial and other material stresses through job loss and underemployment), it became clear early in 2020 that women in violent relationships, and their children, would be at increased risk. Moreover, the organizations designed to serve them would be severely impacted by pandemic-specific and -related factors. Even prior to this health crisis, VAW services were under-funded, limited in their ability to support women in leaving violence due to a chronic lack of safe and affordable housing, and tasked with serving women and children experiencing the acute and longer-term effects or trauma and violence, including mental health and substance use concerns; in some cases, especially in under-served rural areas, women’s shelters also became de facto homeless shelters for women, even if they were not experiencing immediate risk from violence. Adding the COVID-19 pandemic to these longstanding issues created a perfect storm of what we’ve termed “multiple pandemics” [8] starting in 2020 and continuing to the present day.

### Women’s shelters and “rules”^1^

Emergency shelters for women experiencing acute violence from a partner are unique environments. While they have some similarities to other services for those experiencing homelessness, including space constraints, time limits on stays, etc., safety from immediate violence from the abusive partner is the paramount consideration; as well, children are almost always present in these shelters. It is well-established from processes such as domestic violence death reviews and related research that when women leave, or attempt to leave, a violent relationship, or take other steps towards safety for themselves and their children, the risk of severe violence significantly increases. Leaving is, in fact, a key risk indicator for femicide in the context of violent intimate relationships, as the abusive partner begins to feel the loss of control and may lash out to re-establish dominance [9]. Thus, as women’s shelters in Canada evolved from private homes where women could hide, to government-funded facilities with explicit mandates, procedures and accountabilities [10, 11], the emphasis on keeping those inside safe, through features like double, buzz-through steel doors, security cameras, visitor logs, etc., made shelters sometimes feel like prisons designed to keep people in, as well as out.

As noted above, shelters are chronically under-funded and over-capacity [12–14], with physical space designed to maximize beds, not flexibility in space use, and therefore women are routinely triaged for service. Even before the COVID-19 pandemic, shelter leaders and staff balanced rules of stay with client autonomy, woman/child well-being, especially in the context of the traumatic effects of violence experiences, and the safety of staff and other residents [15]. At the micro level, this could play out as rules designed to reduce risk by limiting outings and visitors, enforcing curfews, and requiring abstinence with respect to on-site smoking, alcohol and drug use. At a more macro level, this has meant organizational mandates that explicitly exclude those deemed as “high risk” or “unsafe”, including women actively using certain substances or in acute mental health distress, being excluded from residential services [14]. As Kulkarni et al. [15] note, and we have explored in related analyses [8], these rules often reinforce or re-activate women’s previous experiences of violence, stigma, inequity and structural racism. In the context of severe capacity limits, the issue of who deserves service, and who is “safe” to be in shelter, takes on additional importance, especially as pandemic restrictions began to emerge that further limited access to shelters [16].

### Women’s shelters and public health

There is a paucity of literature on the role of public health regulations, programs or services as they pertain to VAW services. No literature was identified detailing any relationship between local or regional public health authorities, and those providing VAW services. Anecdotal evidence from VAW organizations in Ontario, Canada (our research context), and examination of the prevailing public health legislation, the Ontario Health Protection and Promotion Act [17], reinforced that these two sectors were, prior to the current pandemic, largely siloed. At the individual service level, there may have been occasional referrals by VAW workers of women to specific public health programs, such as well-baby visits or sexual health clinics, and in some cases public health nurses would conduct visits in the shelter, but very little else. For those organizations large enough to have a commercial kitchen, food safety regulations and inspections would apply. In one case, an executive director (ED) partnering on this research commented on a positive relationship with public health that also included advice on infection control practices prior to COVID-19, as well as the above activities, but this was an exception, at least among this anecdotal survey of Ontario VAW agency EDs. The lack of significant organizational-level interaction between these sectors prior to COVID-19 is interesting given VAW’s framing, as noted above, as a “major public health issue” by the WHO and others. This presents a research gap, as well as an ideal space in which to explore how COVID-19 responses impacted a sector rarely previously considered by public health authorities.

The current research, co-led with leaders from the VAW sector in Ontario, Canada, is part of a larger mixed method project designed to understand the impact of the pandemic, and responses to it, on VAW services generally. The present analysis examines how the evolving policy context, as represented by emerging guidance and protocols from public health and other government authorities, impacted those receiving, providing and guiding care in shelters for women experiencing violence. The specific research question was: how did changes in public health and government rules, and how these were communicated, impact the care provided and received in women’s shelters?

## Methods

Interpretive description (ID), combined with an integrated knowledge mobilization (KMb) approach, provided the methodological context for this study. ID was originally conceived as an inductive qualitative approach to provide ways of understanding phenomena that are useful to applied health practice [18]. Integrated KMb is an approach to research that engages knowledge users in the development, implementation, and dissemination of mutually beneficial research [19, 20]. The two approaches are well-aligned in that they prioritize the articulation, through data analysis and meaning-making, of actionable messages that are explicitly embedded in existing social and cultural environments, and reflect both what is known, and what needs to be changed, about practice and policy. In our project, and with a focus on providing pathways for action, we collaborated with five executive directors (EDs) from urban and rural women’s shelters in Ontario, Canada as active partners in crafting the research questions, recruiting participants, and framing how results were communicated to various audiences. The content experts on the research team, who are immersed in relevant literature, complemented the lived expertise of the EDs to ensure, consistent with the ID approach, that extant knowledge was continuously embedded in emerging findings to create recommendations that would both advance practice and policy, while being grounded in existing realities. Further methodological details are described elsewhere [21].

### Sampling & recruitment

Women using emergency residential shelter services, staff providing direct service, and executive directors (EDs) in these and related organizations serving women with violence experiences were eligible to participate. We used purposive and snowball sampling to recruit participants. Research partners supported recruitment by 1) ensuring shelter staff were aware of the study; 2) asking shelter staff to advise women about the study; and 3) inviting ED colleagues from both women’s shelters and other services women may use when experiencing violence (e.g., homeless shelters, trauma counseling agencies) to participate in the study.

### Participants & procedures

The participants in this study were from 24 VAW/shelter agencies across Ontario, Canada and included three separate groups: 1)eight women (mean age 32 years, SD = 11.13) who had lived in a residential women’s shelter during the pandemic, or alternate housing offered by the shelter due to COVID-19 capacity restrictions (i.e., hotel/motel) [21]); 2) 26 direct service staff; and 3) 24 EDs (mean age 48 years, SD = 9.53) from 14 VAW/shelter organizations, and 10 other organizations providing services to women experiencing violence (e.g., counseling agencies, homeless shelters). The characteristics of those providing data for each group are provided in Table 1.

**Table 1:** Participant Characteristics

Characteristic	*n* (%)
**Women receiving services (*N* = 8)**	
Ethnicity (Caucasian)	8 (100)
Born in Canada	7 (87.5)
Canadian citizen	8 (100)
Employed	4 (50)
Children living with them	5 (62.5)
At least high school education	4 (50)
**Direct service staff (*N* = 26)**	
Years in field (10 +)	15 (58)
Years at current organization (10 +)	9 (35)
Gender (female)	26 (100)
Role at women’s shelter or sexual assault center	
Residential counseling	21 (81)
Outreach (includes housing support, court, public education)	3 (12)
Support Services (administrative, custodial, dietary)	2 (8)
Employment status (full-time)	18 (69)
Worked remotely during pandemic	6 (23)
Location (urban)	22 (85)
**Executive Directors (*N* = 24)**	
Ethnicity (Indigenous)	3 (12.5)
Born in Canada	21 (87.5)
Gender (female)	20 (83.3)
Education	12 (50)
Master’s degree	10 (42)
Bachelor’s degree	12 (50)
College	1 (4)
Highschool	1 (4)
Type of organization (women’s shelter)*	14 (58)
Indigenous shelter/organization	2 (8.3)
Rural shelter/organization	10 (41.6)

^*^Other organization types included: homeless shelters, counseling services, child/youth agencies, etc.

One-on-one semi-structured telephone interviews were conducted with women and staff. Five focus groups with EDs, each consisting of four to six participants, took place via videoconference and each was facilitated by two research team members. These focus groups were repeated with the same participants approximately 12–14 months later – i.e., we heard from leaders relatively early in the pandemic (“wave 2” in Ontario in late Summer/early Fall 2020) and later (“wave 3”, Fall 2021, prior to the Omicron variant arriving in Ontario).^2^

Interviews and focus groups addressed questions regarding participants’ service-related experiences during the pandemic. The first set of discussions occurred prior to vaccines, thus focused on the earlier stages of the pandemic, as staff and leaders re-organized their work flows specific to physical distancing, quarantine and infection control guidance (personal protective equipment (PPE), cleaning, etc.). The follow-up focus groups with leaders allowed us to ask about vaccination-related protocols and mandates, and how these unfolded in organizations and across the sector.

### Data coding & analysis

Interviews and focus groups were transcribed verbatim using a professional transcription service and coded using Quirkos qualitative analysis software (Quirkos 2020, Version 2.4.1). Along with the principal investigator, team members involved in conducting the interviews and focus groups developed a preliminary coding scheme. Using this, a subset of transcripts was coded independently, each by two of seven core research team members. Pairs of coders met to discuss the coding scheme and brought suggested revisions back to the larger group. After several rounds of discussion and revision, the coding scheme was finalized, remaining transcripts were coded by two team members each, and the Quirkos files were combined. Two team members assigned to the thematic area specific to public health/government rules read and re-read relevant text to further extract meaning and develop an understanding of the data overall, while also, per the interpretive description approach, recontextualizing participants’ comments within the broader literature [22]. Revisions to the names and definitions of codes were made throughout, as needed. All authors provided input on the quotes presented in this paper and attention was paid to ensure representation of a variety of participant perspectives, as well as to include both converging and diverging points of view. As part of our integrated approach to knowledge mobilization, study partners were consulted on emerging themes during two half-day sessions dedicated to sharing and discussing the interpretation of findings, and how best to communicate them to specific key audiences. Thus, the recommendations presented at the end of this paper were co-developed with the VAW organization EDs partnering on the study.

### Ethics approval and consent to participate

This study was approved by the Western University Non-Medical Research Ethics Board (REB Protocol 115865). Informed consent was obtained from all participants.

## Results

**Fig. 1:**
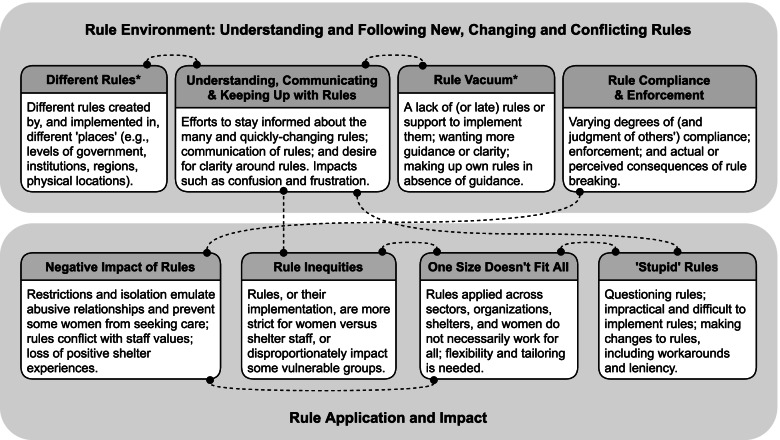
Themes, Theme Descriptions and Conceptual Links Among Themes *Data from all three sources (women and staff interviews, focus groups with executive directors) contributed to all themes except these two which were present in staff interviews and focus groups only

Our analysis identified eight themes that we organized under two domains. Comments from EDs, direct service staff, and women receiving care were often coded under more than one theme. Figure 1 describes the eight themes, conceptual links between them (as indicated by repeated coding overlap), and the participant groups (EDs, staff, and/or women) in which each theme was present.

### Rule environment: understanding and following new, changing, and conflicting rules 

#### Different rules

One of the key themes, present in ED and staff data, was the notion of different rules coming from, and being implemented in, different ‘places.’ For example, some participants commented that rules varied among public spaces, businesses or agencies, and could also vary from one shelter to another, depending on their regional public health unit. An ED described the challenge of balancing rules from various levels of government with expectations from funders:

*And it makes it difficult to be able to give the quality care that we want to give, but we need those spaces because I’ve got Ministry [funders], I’ve got Public Health, I’ve got Ontario, I’ve got like a whole bunch of people telling me that I can’t have them [clients] in shelter, so it makes it a whole lot more challenging. So, when I talk to my funder about like oh, we have this challenge, it’s like managing all of these spaces.* [FG3, time 1]

Or as another ED in a follow-up (Fall 2021) focus group said:

*[W]e’ve been treated like a congregate care setting throughout the entire pandemic… so when they started opportunities to vaccinate … other public health units in other parts of the province were qualifying VAW shelter workers as healthcare workers and interpreting the direction from the Ministry of Health as this includes the shelter workers. But in [location] that wasn’t the case and so it got pretty fierce for a few days, [we] had some pretty intense conversations with Public Health and it didn’t make a whole lot of sense… what I learned is it was an inconsistent interpretation of what the Ministry was saying to public health units.* [FG5, time 2]

Rules created at higher levels were often not specific to the shelter context, therefore EDs and staff engaged in a process of deciding which rules to follow, i.e., which were most applicable and aligned with shelter practice, and where additional information might be sought to support these decisions. For example:

*But we didn’t go with the health unit’s version [for guidance for developing vaccination policy] because one of the neighbourhood resource centres hired a lawyer to develop a policy that was well suited to the work that we do* [FG5, time 2]

Moreover, this staff member described wanting to be more involved in how to implement rules and not even knowing where various rules came from:

*…I guess the policies got put in place and then it was kind of up to us as workers to relay to our women… because I don’t know if some of them are coming from – like what is coming from the government, health units, and then what is coming from the agency itself. I think that I would like more clari[ty]… because you can’t do anything about changing the government’s rules or policies, or even the health unit... we can ask questions or kind of say, the impact of what the agency's policies may have on the women… [I want] to be able to have that dialogue around that*. [S13]

### Understanding, communicating & keeping up with rules

Women, staff, and EDs shared comments about understanding and communicating rules during the pandemic. For example, EDs described the difficulty of staying up to date:

*Just really having to really educate myself and keep in tune with what is happening on a daily basis on the news with COVID and just the changes … occurring with bylaws and different policies that we have to add to, you know, the policies and procedures to our shelter to ensure the safety of our clients, the staff....* [FG5, time 1]

Challenges related to understanding rules also had to do with the pace at which rules changed:

*I think some of the challenge in all of this has been discrepancy in information and what seems like misinformation because you’re given one directive or certain information one week and then the next week it’s quite different and I think the entire population has experienced a bit of that. So that has made it challenging for educating our staff so that they have knowledge so that they’re then able to support clients…. Particularly at the beginning, it was changing all the time. So trying to be flexible and taking in new information and that constant change can be challenging.* [S23]

There were also challenges related to the communication of rules within shelter, and to the shelter from outside sources. This included EDs navigating how to relay important information about government mandates and pandemic protocols from a number of agencies to their teams without inundating them with an overwhelming amount of information that sometimes changed drastically throughout the day. Others noted that communication from public health and government authorities often included over-explanation, with sometimes redundant links to outdated guidance, or information tangential to the core message; as one ED said: “*break it down to what do we need to do. Six items, what do you expect us to do. Not 15 pages of there’s this option and there’s this, and there’s this. What are the bottom lines, what are your expectations?*” [FG3, time 2].

The abundance and ever-changing nature of rules led to confusion for everyone, with EDs commenting on a “trickle down” effect of the lack of clarity from external authorities impacting their ability to guide managers and direct service staff, and from there, the ability of staff to best support women. The following series of quotes exemplifies this:

*When you’ve got people that are moving around with no clarity with regards to what’s your portfolio now and what does this mean, like that’s challenging. So clarity from the government with regards to who’s on this team, what are their different roles. How is Public Health and the Ministry intersecting because, you know, there were times where I asked the Ministry questions, they were like – they’re trying to figure it out themselves and, you know, they don’t know who these people are* [FG1, time 2]

*…But sometimes our feedback like from management is… there’s been a lot of like contradictory things and so it just doesn’t feel very settled, I’ll put it that way… but I will say overall, it seems that all of us are somewhat confused about what we should be and what we should not be doing*. [S2]

*I had a few little problems there with understanding some of the rules… We did a meeting about once a week, sometimes two times [for] COVID updates, and I would ask for the form so I know the rules, so I know everything, and they would tell me that I’m not allowed to have documents because it’s something to do with if they’re service documents that I can’t, and we’re not allowed to have one. So, it was a little difficult to understand the rules and stuff.* [Woman (W) 5]

At the point of care, staff were aware that clients wanted more information and wanted to communicate more clearly but faced barriers in doing so. For example, women identified that the way in which restrictions were delivered or communicated to residents could have been improved and feedback could have been sought from clients, where possible. Staff tried to be as transparent as possible with clients, even amidst changing rules and mandates that were difficult to understand and employed a trauma- and violence-informed approach wherever possible to deliver, in compassionate ways, difficult information about isolation requirements and virus updates.

### Rule vacuum

While at different times during the pandemic there was an (over)abundance of rules, at other times, or with regard to certain issues, staff and EDs experienced a ‘rule vacuum.’ For example, this ED spoke about not having guidance when they needed it:

*So, I went ahead and built a policy around what we were going to do in-house and how I was going to implement it. And thank God it was in line with – partially at least – with what the Ministry wanted to implement in June. But I didn’t know. I had no idea. So, I felt like OK, we’re spinning our wheels doing all this stuff when… I like the idea of having some autonomy, but on the other hand, it would have been nice to give us some kind of a framework for what expectations [they] had of us.* [FG4, time 1]

While EDs sometimes lacked guidance from their local public health authority or from various government Ministries, at the shelter level, some staff members spoke about particular situations where there were no rules in place for what to do, for example, if/when: a shelter client got COVID-19, staff were in contact with someone who was in contact with a positive case, or there was a delay in receiving clients’ COVID-19 test results. This staff member felt a need for more and clearer rules in general, while recognizing why this wasn’t always possible:

*…but if we had really clear rules and boundaries and expectations, I think that would actually be super helpful. I would definitely appreciate more of that. But I know that’s also hard, because things come up and you just have to wing it because you haven’t experienced it. Right?* [S2]

A lack of rules, or guidance for how to implement them, resulted in extra work for EDs and staff, but also tremendous stress and frustration. This ED described needing to figure things out on their own:

*I felt pretty hung out to dry for many, many months and we finally just stopped asking them for help and we went and found our own guidelines, our own tools. And then by the time [area] Public Health was getting out how we’re supposed to be running things, we’d already put all these practices into place and found money and found, you know, donations… like I’ve never in my life felt so much anger and frustration and feeling so abandoned by a public health unit that was supposed to be protecting us.* [FG2, time 1]

This extra work and frustration associated with the lack of guidance remained a common theme in the follow-up focus groups.

Finally, staff and EDs also described how, even when there were rules, they did not necessarily come with the information needed for implementation – guidance had to be tailored to the shelter context.

*[Shelter management] asked us our opinion with everything, but mainly, it came from, like, public health. So, anything that … the Ministry or public health would mandate, then we would have to obviously follow, right? So, they would put it in place, and then our manager would ask us, like, ‘What is your opinion? How do you think this is going to work?’ or, ‘What are some ways that we could do it?’ And because we work so closely with the women and are always in the hub of everything, we do have more information that would work better, right?* [S25]

### Rule compliance & enforcement

Given the number of new pandemic-related rules added to the pre-existing rules for staying in shelter, issues of compliance and enforcement were never far from the surface. For example, this ED wrestled with complying with COVID-19 rules in shelter that went against her values for providing care and, in her view, compromised women’s safety:

*When I get stuck on [whether to comply] and start thinking I should follow some of the compliance guidelines, I sit down and I say to myself, and I have done this four times in the pandemic: if this woman dies, and these are four specific women, what will I be able to say on the stand of an inquest that makes any sense? Will I say I followed these rules and therefore she had to go here and leave here and not have the safety that she needed in that acute moment?* [FG1, time 1]

Again, this was an issue that persisted across time. EDs in our follow-up focus groups also discussed rules that did not prioritize women’s safety and were facing new issues when it came to vaccination enforcement for clients in shelter: “*If it's a choice between a woman being homeless or living in danger or living rough, and coming here and having to be vaccinated, the choice is very easy for me.*” [FG2, time 2].^3^

Many direct service staff were concerned about enforcing rules rather than prioritizing women’s autonomy, and felt challenged regarding whether, and how, to enforce the rules. For example, this staff member was able to rationalize enforcing isolation requirements for women in shelter:

*So we did it because respecting health guidelines and trying to respond to a pandemic takes precedence over personal choice and freedom to make your own decisions. So we did come down pretty strongly with that and if people were not able to maintain that, we told them that they needed to find another place to be.* [S22]

This staff member described the varying reactions that women had to staff members’ enforcement efforts:

[I]n the beginning it felt like we were really policing people because we were constantly like… oh no, you can’t be hugging … your mask, you know… don’t forget to sanitize when you get back in, like we were constantly like just reminding people. And, you know, because people were under a lot of stress sometimes people were like ‘oh, OK sorry,’ you know, and sometimes people were like ‘are you f*cking serious?’ [S16]

Overall, staff enforced rules to varying degrees, and some mentioned that it was difficult or impossible to do where children were concerned (e.g., ensuring young children maintain physical distance in shelter). Moreover, there were varying levels of compliance (e.g., related to mask-wearing) among and between staff and women that sometimes led to tensions in the shelter. One woman described how she felt when another woman in shelter was not being compliant: “*And the other family just – she doesn’t follow the rules, so she kind of does her own thing. Like to me it actually pissed me off. And I complained”* [W6]. In contrast, some women strictly adhered to the rules, for example, this staff member spoke of a woman who was supposed to stay in her room: *“… that’s how restricted she felt, that her daughter had fallen and was bleeding from her mouth, and she still didn’t leave the room to come out to us*” [S5]. Finally, some women spoke of the actual or suspected consequences of breaking the rules. One woman described being “*called up*” (i.e., reprimanded) for getting too close to other people. Another woman said,

*A lot of people… knew they had nowhere else to go… so they wouldn’t question [the rules]… and they were afraid they were going to get locked in or… or everyone was really scared of getting in trouble, like and we were going to get kicked out and then be on the street*. [W5]

### Rule application & impact

#### Negative impacts of rules

In general, COVID-19-related rules made shelter work and life more difficult for all involved:

*We can’t just make decisions just based off of a pandemic. Like we can get creative because it’s not working for the women. And then it’s making staff’s job harder because we’re trying to navigate ways to support them when we’re like so limited with what we actually can do if we follow all the rules.* [S21]

Later in the pandemic, EDs particularly struggled with staffing issues caused by rules, for example:

*I’ll also mention… the mandated policy that we got from ministry also required us to have all of our volunteers be vaccinated or [have regular testing], so we lost about 30% of our volunteers… [volunteers] answer our crisis and support line.* [FG5, time 2]

As this staff member described, there were serious concerns about the impact of service changes to accommodate new rules on quality of care: “*Some of the orders and some of the directives really, from a trauma-and violence-informed perspective, didn’t necessarily always meet the needs of the women that we were supporting*” [S20]. In addition, the rules felt so restrictive for some women that they left shelter (to return to their partner or other unsafe space) or were reluctant to come at all. As this woman commented, at times the rules seemed to take priority over women’s best interests: *“… and it almost feels like bullying at some point where it’s just they’re not – like they’re taking the rules more seriously than like the community’s benefits and the mental side of things*” [W5].

The language of not being “allowed” to do various things was quite common throughout the data. Perhaps even more troubling was how women, staff, and EDs spoke about the controlling aspect of rules (e.g., not being allowed to leave one’s room, limited connection with staff or other residents) and how they replicated the abusive relationship dynamics that women were trying to leave behind. For example, this staff member described the requirement that women were accompanied when they left the shelter for errands or appointments:

*It’s created a difficult situation, and women have been upset. It ties back to feeling like they don’t have that control. And I’m sure that that is something that they’ve already experienced so often in life, fleeing domestic violence. So to now have to have that control over when they leave and when they come on our timeline has been really difficult.* [S19]

Accordingly, staff and EDs were negatively impacted when they felt pressure to, or actually did, implement or enforce rules that compromised their values:

*It also continues to be a challenge because of some of the directions we’re getting from our Ministries about some of the rules and expectations that we have to follow. They’re not trauma- and violence-informed. They’re just not. And some of those expectations are at – there’s conflict there… when the Ministry is holding the purse strings and then you having to comply when it feels like it’s a violation of some of your organization’s values, it’s a tricky space to navigate.* [FG3]

Again, this sense did not improve as the pandemic evolved to the point of vaccines being available, though there was far more diversity in opinions on this specific issue, with, as noted above, vaccine mandates being applied to staff, but not to women using services:

*…we’ve always advocated for people and women’s bodies not to be policed by other systems, right. So how is it that we are supporting the government’s decision that people need to be vaccinated in order to maintain employment, how is it that we’ve jumped onto that so well and are willing to give up a core principle and value that we’ve held for so many years?* [FG1, time 2]

Finally, some of the negative impacts of the rules took the form of the removal of positive experiences. Specifically, use of communal spaces, group activities, and physical contact were not allowed. This participant described what it was like:

*And it was just so, like, we’d set up lawn chairs in a parking lot for people to sit out there. And their family would come, they were not allowed to hug them, they’re not allowed to, like, you know, when you see your mom, you want to give her a hug. You can’t do that. You’re going through this traumatic experience where you’re torn away from your family and your home and everything, and these grandkids can’t run up and hug their grandmother, right? ... It’s heartbreaking to see.* [S25]

### Rule inequities

Women, EDs and, particularly, staff, spoke about how the rules, their implementation, or their impacts were not always fair. There were two main ways these inequities took place, with rules disproportionately impacting 1) clients (as opposed to staff), and 2) vulnerable groups. For example,

*We’re saying to women that they’re not allowed to go out and they’re not allowed to talk to each other. But we have the privilege to do that in our – to whatever, in our home life, so would have to be mindful that how we enforce those rules to the women that have no choice, would have to be conscious of that and we need to take that in consideration when we are out and about with family and friends and interacting too as well…* [S15]

In contrast, one staff member pointed out that the inequity sometimes went the other way, with women in shelter being allowed to do things after their initial two-week quarantine,^4^ such as leaving the shelter over-night, that staff did not feel free to do themselves in the interest of keeping women safe.

Some women using shelter services were particularly impacted by rules that no longer allowed care to be tailored to their unique needs. Those described as experiencing inequity because of rules (within and/or outside of shelter) included Indigenous women and women of colour, as well as women with low literacy, those on low income (e.g., who can’t afford masks or hand sanitizer), with mental health concerns (including anxiety related to mask-wearing), and women who are not fluent in English. This staff member described a situation where one woman was disproportionately impacted by isolation and how shelter staff tried to help her by bending the rules:

*But she went right from isolation in her own home to isolation in a completely new place where she knew absolutely no one. She doesn’t speak English fluently. And so, she was having, like, complete mental health crises during the quarantine period. And, we couldn’t do anything for her, you know. Like, we were so restricted. And, you know, we problem-solved constantly… We eventually settled on allowing her… and her daughter to go… [to] a courtyard in the middle… which has a small playground for really small kids in it. And so, we ended up telling everyone else that they couldn’t go into the courtyard and she was allowed to use the courtyard for the second week. So, that helped her.* [S5]

These staff members described how women of colour were less able to advocate to have their needs met and experienced discrimination with regard to rule enforcement:

*And for me as staff, it’s so hurtful. As a Black woman, it’s so hurtful to see how when you know [how] to navigate the system and challenge how you get your needs met… those women of colour who are so fearful and just kind of say yes and just go along with whatever.* [S14]

*And I’ve heard from Indigenous members from the community that are saying if I was to require [vaccine mandates], I wouldn’t have any staff, right, because my community doesn’t trust the government or anything the government does. We’ve seen genocide, we've seen… and I could say the same from the Black community, right?* [FG1, time 2]

### One size doesn’t fit all

One of the reasons the rules didn’t work well for women, staff, and EDs was that they were often ‘blanket’ rules to be implemented across sectors, organizations, and clients, and/or across an entire type of setting, such as “congregate care”, despite significant heterogeneity between, for example, a long-term care facility, a penitentiary, and a women’s shelter. Some participants spoke about it being unclear how rules created at a higher level (e.g., by the funding Ministry) applied or should be implemented in the VAW shelter context, or that it didn’t make sense to have the same rules apply to both long-term care homes and shelters. More often, however, this theme was present at the individual level. For example, this staff member contextualized the rules within the VAW sector that is known for providing care based on individual women’s needs:

*It’s hard. It’s hard. It’s been really difficult, because I’m just like scrolling through who has been in and each woman is so different and has different needs, that how they’re handling COVID is also really different. There’s always challenges. It depends on what they’re going through.* [S24]

For women, this theme was present in their comments about the need for flexibility or accommodation.

*I think there could have been sort of more meeting in the middle in terms of just mental health and letting us outside for a little bit… There was no real discussion about what would benefit us, it was just kind of, like, okay, this is what’s happening. And I understand the seriousness of it all, but considering that we were coming from traumatized recent pasts and the need for mental health [support] in that case… And just, like, a little bit of sunshine can do enormous things for your body and mind and soul, so yeah, just a little bit more flexibility in that regard alone would have made a huge difference.* [W2]

### ‘Stupid’ rules: applying judgement and workarounds

The most common theme across the interviews (especially among staff) and focus groups involved participants questioning the rules (e.g., pointing out inconsistencies), highlighting the impractical nature of rules or the difficulty to implement them, or describing ‘workarounds,’ including developing new processes, or adapting, breaking, or loosening rules. For example, this staff member disagreed with specific rules related to COVID-19 testing:

*But I don’t understand our logic, if a woman comes in and she’s staying more than two weeks, then she’ll need to be tested for COVID [after] two weeks. And I’m like well that doesn’t make sense, why don’t we get her tested when she comes in, because if she has COVID, I don’t want to find out two weeks later. That’s something I don’t agree with in our policy, I think that’s rather silly.* [S11]

Furthermore, these staff members described how some rules were impractical for a shelter setting: “*when you have a four-year-old who… literally all she wants to do is hug everyone and play with everyone, you cannot tell a four-year-old to social distance. Like, it just doesn’t work*” [S5]. Similarly, this ED described how some rules were difficult to implement and the “fight” it took to make authorities understand: “*So we fixed that [vaccine requirement for everyone on building entry], that’s no longer the case anymore but it was a fight that just – it took energy that we didn’t really have”* [FG5, time 2].

Finally, these participants spoke about how they needed to do what they felt needed doing to support their clients:

*We really can’t have clients in the office. That was something that was stressed in the beginning and still is. Due to the nature of the work, we’ve had to, like, have some leniencies on that. But… I have a chair in front of my office and my desk, and I used to have clients just sit there, we’d talk, we’d work on things.* [S18]

*When a woman comes into shelter they are to be 14 days in isolation. Yeah. And some shelters like it didn’t make sense it was increasing the risk to women so we chose not to do it. But then we’re going exactly against to what the Ministry was directing us to do. So when you gave that feedback [to the Ministry] there wasn’t any change or movement ... So then you have to go against what they’re saying because we know it’s not going to work to keep women safe, it’s going to increase their risk.* [FG3, time 2]

### Summary

Dealing with COVID-19 rules, trying to fit them to the context of residential care for highly traumatized women and children, while trying to manage the impacts of the rules on services, was frustrating and exhausting. In our follow-up focus groups with leaders, we heard about the profound exhaustion they all felt, and the loss of many in their ranks, either to early retirement, or to sectors that pay better and are less fraught – both in the type of work, and in the constraints put around it. The following quote provides an excellent macro-level insight into the disconnect between how, why and where rules originated, and the harm that a lack of understanding of the work of women’s shelters among public health and government authorities had on care:

*We are talking about the impacts on policy, on government, and our workload around policy and keeping women safe; to me, the issues that are being missed are what’s changed for the women, how much higher risk they’re at. Why is the Ministry not asking that? What are you seeing differently? What do we need to give you to keep women safer? What are the issues on a larger scale that have changed and shifted? We’re talking policy, we’re not talking about keeping women alive. And I find that very frustrating when that’s the embedded piece of our work, that’s where my heart is, and I'm being forced not to focus [on that] because now I don’t have the damn time to focus on what I feel matters more, because of these other things happening up here at this policy/governmental level. And then things* – *the real reason of why they fund us and why we exist in the first place, let’s take a look at that.* [FG3, time 2]

## Discussion

The present analysis highlighted how the evolving rules environment during the first 18 months of the COVID-19 pandemic impacted service delivery to women experiencing violence. As for many health and social service sectors and organizations, inconsistency and lack of clarity in COVID-19 specific and related rules, and how they were communicated, was frustrating to all involved – leaders, staff and those receiving service – and a source of significant stress, and increasingly, exhaustion. In VAW services, however, these consequences played out in uniquely problematic ways, especially in the context of the intense, highly relational counseling, system navigation and support work undertaken within usually crowded shelters, where highly traumatized women and children find safety, begin to heal and start looking ahead to next steps. For example, when specific COVID-19 regulations, such as 14-day in-room quarantine upon arrival, were implemented, especially in precautionary ways rather than for actual outbreaks (which were very rare in Ontario’s women’s shelters), the balance between “safety from Covid-19” and “safety from violence and its traumatic effects” was lost. Isolating women in this way directly replicated a common tactic of coercively controlling partners, and everyone involved, especially staff, knew this was harmful. In a related analysis, we discuss this in more detail [23] and frame these harmful practices as both vicarious trauma and moral distress.

In the absence of consistent guidance on pandemic rules and how to implement them, many shelters filled this vacuum with creative, shelter-specific solutions to re-instate the balance noted above; this sometimes involved finding ways to work around or tailor rules to reduce harm to women and children. However, this was difficult work, compounded by the fact that unlike most health and social services, VAW services in Ontario are funded by multiple provincial Ministries^5^ (primarily the Ministry of Community, Children and Social Services, but also the Ministry of the Attorney General), each with different accountabilities. During the pandemic, additional rules flowed from the Ministry of Health (MoH), sometimes directly, and sometimes via MoH’s direction to local public health units, which themselves are governed via both provincial and municipal mechanisms (i.e., legislation, Boards of Health) and funding agreements. Compounding this were federal guidelines, which while not mandatory, were part of the broader information environment, adding another point of potential confusion. Shelters often also rely on donations to fund operations, thus they also consider themselves accountable to their local communities, as well as to their feminist principles, including as advocates for women’s autonomy.

As in the broader public, there was often the perception within the shelter that the rules did not apply equally to everyone. In our data, this was expressed as a concern for women of colour, Indigenous women, or those whose first language was not English; a finding we also report in a companion analysis [8], and which emerged in an analysis from Norway, which also found differential impacts of COVID-19 on marginalized groups seeking shelter [24]. Similarly, staff felt that decisions regarding such things as who works from home, and who takes up preparing and delivering meals to women’s rooms, were not always clear, or equitable [23].

As noted above, most VAW organizations, at least in the Ontario context, had very little interaction with public health, especially at an organizational level, prior to the current pandemic. While there was some referral of individual women and children to public health programs, and in some cases onsite nurse visits, it was rare for there to be formal interactions at the leadership level, or, for example, at community coordinating tables. This lack of familiarity, in both directions, likely exacerbated the confusion among, and the negative impacts of COVID-19 regulations on, VAW services. We heard repeatedly in this data, and companion analyses, that “one size doesn’t fit all” and that regulations treating VAW shelters like other congregate settings, from long-term care to prisons, did significant harm to women, and hindered their ability to provide trauma- and violence-informed, culturally safe and equitable care. In addition, while most shelters involved in this study had emergency, and in some cases pandemic-specific, preparedness plans (usually arising from the 2009 H1N1 influenza pandemic), these were not dictated by public health, and in fact were often made moot by the deluge of COVID-19 rules coming from the various authorities.

### Limitations and future research

Due to the ever-changing nature of the COVID-19 guidance being provided by multiple government and public health agencies to the participating organizations (and the VAW sector more broadly), the time frame of our data collection and analysis (over approximately 14 months), and the overall number of organizations represented in the data (24), we did not analyze the specific protocols that each participating organization was using at any given point in time, relying instead on their own experience of the myriad rules being provided, and their interpretation of how to apply them to their services. In a separate sub-study from this project examining how these regulations impacted the physical space use of shelters [25], it was clear that certain protocols were more restrictive than others, but that flexible interpretation of these could mitigate the impacts on service delivery.

Future research could examine in more depth the impact of vaccine mandates on the service environment, especially from the perspective of women using services. While Ontario’s shelters were never mandated to impose vaccine requirements for access to any form of service, including residential shelter service, community perception may have influenced women’s decisions to seek care. Staff vaccine mandates also varied, or were applied in different ways, another area warranting examination.

### Implications for practice and policy

Given our integrated knowledge mobilization approach, and our use of interpretive description as an action-oriented methodology, we co-developed, with our VAW organization partners, a number of recommendations for policy and practice specific to developing and communicating rules and regulations during a crisis. These are summarized in Table 2.

**Table 2:** Summary of Recommendations for Policy & Practice

Policy-focused recommendations 1. VAW services have multiple government and non-government funders. Getting different, sometimes conflicting rules, from these Ministries, along with local public health guidance, is unduly confusing and a barrier to developing consistent protocols •One size does not fit all: While women’s shelters are congregate housing, they are very different from others in terms of services provided, size, type and age of facilities. Given their unique contexts and clients, each agency should be supported in tailoring protocols to their specific needs to minimize harm to women and children while respecting health and safety •Protocols and rules must be synchronized across Ministries and Public Health, and aligned with both regional contexts, and the specific context of VAW services, ideally in consultation with VAW sector leaders 2. Any new rules or protocols require clear and timely communication and resources, including time, to support implementation
Practice-focused recommendations 1. Inconsistency or lack of clarity in rules, and how they are communicated, is frustrating and a source of significant stress for women using shelter services, and staff •Women need to understand what options are available (e.g., in shelter, alternative housing, and in outreach) and what’s expected of them and their children in these spaces. These must be clearly provided taking into account various literacy factors •Staff need information and support so that they can effectively communicate and explain rules to women 2. As in the broader public, there was often the perception that the rules did not apply equally to everyone, such as women of colour, Indigenous women, and/or those whose first language was not English •Sector leaders need to provide oversight to ensure that rules are applied equitably, including being clear why different rules might apply to women versus staff, or between different staff groups

## Conclusions

Women’s shelters during “normal” times are generally high rules environments [14, 15], and public health rules imposed during the COVID-19 pandemic added to the existing and daily negotiations between and among staff and women, highlighting existing tensions, such as the inequities noted above, and revealing new ones. To the extent possible, and in future crisis situations, rules must be synchronized across government funders and public health authorities, and aligned with both regional contexts, and the specific context of VAW services, ideally in consultation with VAW sector leaders, which has been lacking. The work of synchronization should be done by the authorities, and not left to each sector or organization to have to interpret diverse and sometimes conflicting guidelines. This leads to both lack of compliance, but also loss of trust, and as a worst case, poorer infection control and a lack of woman-centred, trauma- and violence-informed service provision.

## Data Availability

The datasets generated and/or analysed during the current study are not publicly available due confidentiality provisions in the consent process, but are available from the corresponding author on reasonable request.

## Abbreviations

ED: Executive director
FG: Focus group
ID: Interpretive description
KMb: Knowledge mobilization
MoH: Ministry of Health
PPE: Personal protective equipment
S: Staff
UN: United Nations
VAW: Violence against women
WHO: World Health Organization
W: Woman

## Footnotes

1 For the purposes of this paper, we use the term ‘rules’ to apply broadly to any written/spoken statements or unwritten/unspoken understandings regarding what should and should not be done with regard to COVID-19 at any level, ranging from individual shelter-level policies or procedures to government-level legislation, and everything inbetween.

2 The groups were not conducted in the same order, so numbering of quotes, below, is temporal and does not indicate the same people were in “FG3” at each time point.

3 In fact, vaccine mandates for clients were not, at least by time of writing, put in place for those seeking service.

4 Early in the pandemic, women and their children were required to self-isolate in their rooms for their initial two weeks in the shelter to avoid spreading infections they might have had on entry.

5 During the pandemic, the Federal government also provided one-time funds to support specific shelter activities, and, in non-pandemic times, supports “bricks and mortar” resources via the Canada Mortgage and Housing Corporation.
